# Supplementary figures and images for: Effect of Particulate Matter Exposure on Respiratory Health of e-Waste Workers at Agbogbloshie, Accra, Ghana
Source: Int J Environ Res Public Health. 2020 Apr 27;17(9):3042. doi: 10.3390/ijerph17093042 (PMC7246629; doi:10.3390/ijerph17093042)

# Graphical Abstract

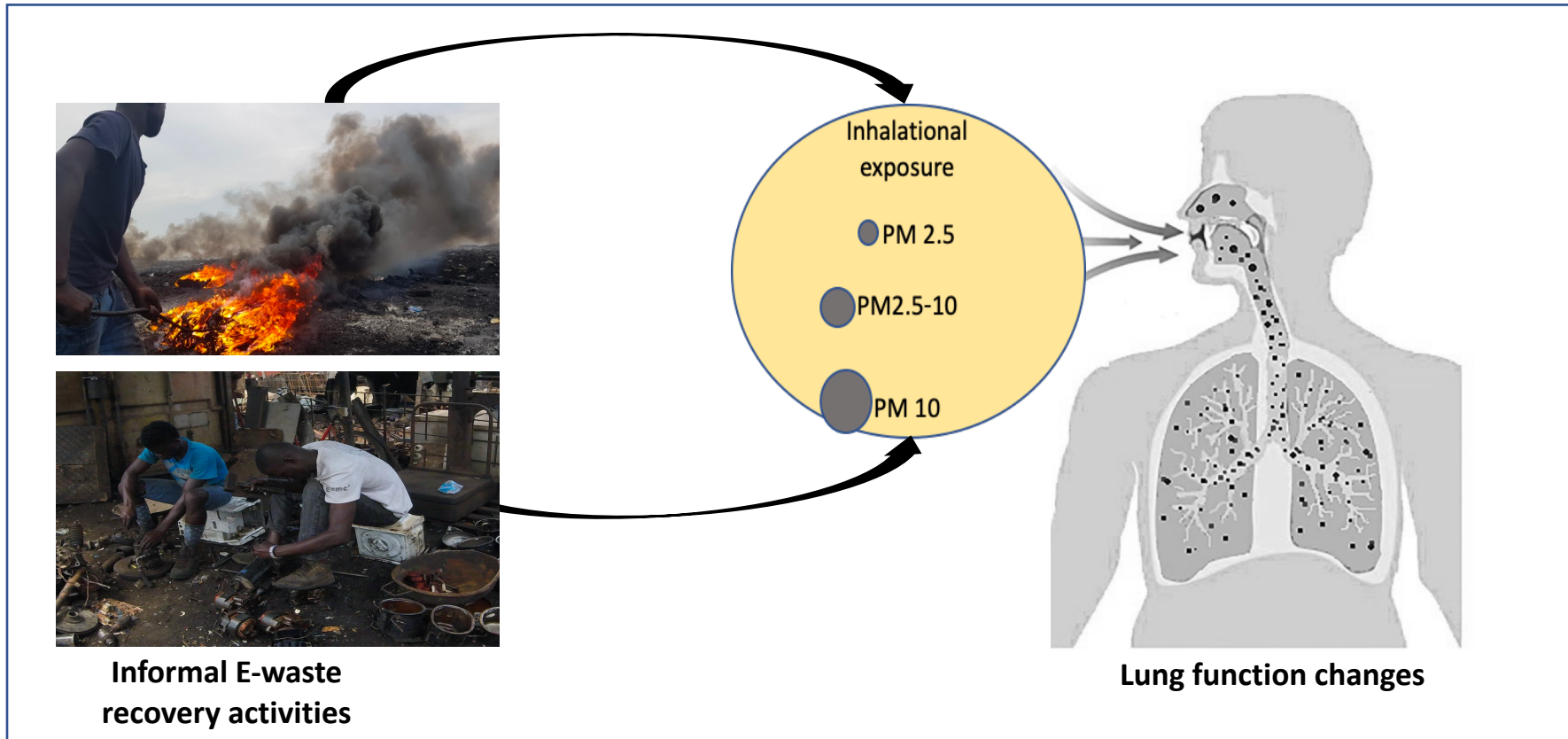

Supplement: Supplementary file 1 [file ijerph-17-03042-s001.pdf]
